# Possible Catch-Up Developmental Trajectories for Children with Mild Developmental Delay Caused by *NAA15* Pathogenic Variants

**DOI:** 10.3390/genes13030536

**Published:** 2022-03-18

**Authors:** Yu Tian, Hua Xie, Shenghai Yang, Shaofang Shangguan, Jianhong Wang, Chunhua Jin, Yu Zhang, Xiaodai Cui, Yanyu Lyu, Xiaoli Chen, Lin Wang

**Affiliations:** 1 Department of Child Health Care, Children’s Hospital, Capital Institute of Pediatrics, Beijing 100020, China; yut262@126.com (Y.T.); child811117@163.com (J.W.); jinchunhuabj@sina.com (C.J.); 2 Department of Medical Genetics, Capital Institute of Pediatrics, Beijing 100020, China; xiehua2005@126.com (H.X.); canon-g5@163.com (S.S.); 3 Department of Neurology, Children’s Hospital, Capital Institute of Pediatrics, Beijing 100020, China; yangshenghai2003@163.com; 4 Department of Laboratory Center, Capital Institute of Pediatrics, Beijing 100020, China; zangyu0612@sohu.com (Y.Z.); xdaicui@126.com (X.C.); lvyanyu@sina.com (Y.L.)

**Keywords:** *NAA15*, novel variant, developmental delay, possible catch-up, developmental trajectory

## Abstract

Variants in *NAA15* are closely related to neurodevelopmental disorders (NDDs). In this study, we investigated the spectrum and clinical features of *NAA15* variants in a Chinese NDD cohort of 769 children. Four novel *NAA15* pathogenic variants were detected by whole-exome sequencing, including three de novo variants and one maternal variant. The in vitro minigene splicing assay confirmed one noncanonical splicing variant (c.1410+5G>C), which resulted in abnormal mRNA splicing. All affected children presented mild developmental delay, and catch-up trajectories were noted in three patients based on their developmental scores at different ages. Meanwhile, the literature review also showed that half of the reported patients with *NAA15* variants presented mild/moderate developmental delay or intellectual disability, and possible catch-up sign was indicated for three affected patients. Taken together, our study expanded the spectrum of *NAA15* variants in NDD patients. The affected patients presented mild developmental delay, and possible catch-up developmental trajectories were suggested. Studying the natural neurodevelopmental trajectories of NDD patients with pathogenic variants and their benefits from physical rehabilitations are needed in the future for precise genetic counseling and clinical management.

## 1. Introduction

Developmental delay (DD) is defined as a significant delay in two or more developmental domains, including gross/fine motor, speech/language, cognitive, social/personal, and activities of daily living [1,2]. DD is reserved for younger children (younger than 5 years), whereas intellectual disability (ID) is applied to older children when IQ testing is more valid and reliable [2]. DD and ID are common pediatric conditions that affect up to 3% of the pediatric population [2,3].

The N-acetyltransferase (NatA) complex, which is composed of the core components of NAA10, NAA15, and other subunits, is essential for regulating multiple cellular functions in humans, such as protein half-life, interaction, and localization [4,5,6]. The *NAA15* gene, also known as N-α-acetyltransferase 15 (MIM 608000), encodes an 866 amino acid protein (101 kD) containing four tetratricopeptide repeat domains and a putative bipartite nuclear localization signal [7]. NAA15 acts as the auxiliary subunit binding with NAA10 and localizes to the ribosome to serve as an N-terminal acetyltransferase [8]. In 2013, likely gene disrupting (LGD) variants of *NAA15* were reported from two patients with congenital heart disease (CHD), and one of them presented additional neurodevelopmental disorders (NDDs) [9]. Subsequently, *NAA15* variants were detected in other independent patients with NDDs [10,11]. In 2018, 38 NDD individuals with 25 different de novo or inherited variants in *NAA15* were summarized. The NDD features of *NAA15* variants are variable and include DD/ID, autism spectrum disorder (ASD), attention deficit hyperactivity disorder (ADHD), and motor and language disorders [12].

In the current study, we identified four novel pathogenic variants in *NAA15* from a Chinese NDD children cohort that updated the genetic variant list of *NAA15*. All affected children presented mild DD after birth, and catch-up trajectories were noted for three of our patients. Meanwhile, the clinical manifestations of all reported patients with *NAA15* variants were retrieved from the literature to confirm their development trajectories.

## 2. Materials and Methods

### 2.1. Ethical Compliance

This study was approved by the ethics committee of Children’s Hospital, Capital Institute of Pediatrics (SHERLL 2020001), and written informed consent was obtained from all the parents for the publication of this clinical information.

### 2.2. Subjects

A total of 769 affected children with NDDs, including ID, DD, ASD, and language disorders, were recruited from the Children’s Hospital, Capital Institute of Pediatrics. Patients with known environmental etiology or other definite genetic causation were excluded. All children had undergone karyotyping, urine/serum metabolic screening, *FMR1* repeat testing, and CNV-Seq to exclude known genetic and metabolic disorders. The clinical evaluations, including physical examinations and neurodevelopmental assessments, were carried out by pediatric neurologists or developmental specialists.

### 2.3. Neurodevelopmental Evaluation

The neurodevelopmental quotient (DQ) was evaluated using the Children Neuropsychological and Behavior Scale-Revision 2016 (CNBS-R2016) [13] or Ages and Stages Questionnaires (ASQ) [14]. The CNBS-R2016 has been routinely applied in China to assess the developmental level of children aged 0–6 years, and it includes five subscales: gross motor, fine motor, language, personal–social, and adaptive behavior. A quotient of less than 70 points indicates developmental delay, and a quotient of more than 80 points indicates normal development. Point values between 70 and 79 are labeled “gray” and require further follow-up and evaluation [13]. For the ASQ, the cutoff scores for the five domains differed according to different age groups [14].

### 2.4. Whole-Exome Sequencing (WES) and Variant Analysis

Peripheral blood samples were obtained from NDD patients and their parents. Genomic DNA was isolated from peripheral blood using a DNeasy Blood and Tissue Kit (Qiagen, Hilden, Germany). Library capture and construction were performed with the SureSelect XT2 Library Prep Kit (Agilent, Santa Clara, CA, USA) and V6 Enrichment Kit (Agilent, Santa Clara, CA, USA) according to the manufacturer’s instructions, and appropriate amounts of enriched DNA libraries were sequenced on an Illumina NovaSeq 6000 (Illumina, San Diego, CA, USA) with 150 base paired-end reads.

After excluding the common variants (1% of the public databases such as dbSNP, 1000 Genomes Project and gnomAD), the candidate variants in NDD genes were retained using the QIAGEN Clinical Insight (QCI) Interpret Translational tool (https://apps.qiagenbioinformatics.cn/, accessed 20 February 2022). The functional effects of missense mutations were predicted by four algorithms (PolyPhen, Sorting Intolerant from Tolerant, Protein Analysis Through Evolutionary Relationships, and Pathogenic Mutation Prediction). The inheritance of candidate variants was validated in core family members via Sanger sequencing. The pathogenicity of each variant was interpreted according to the guidelines of the American College of Medical Genetics and Genomics (ACMG) [15].

### 2.5. Functional Study of the Putative Splicing Variant in NAA15

**Figure 1:**
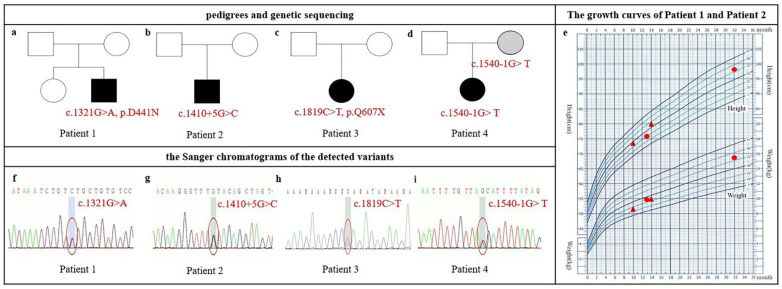
Pedigrees, physical growth curves, and Sanger chromatograms of Chinese patients with pathogenic *NAA15* variants. (**a**–**d**) Pedigree. NDD-affected individuals are indicated by solid squares (male) or solid circles (female). Patient 4’s mother is labeled gray because her intelligence is not available. (**e**) Growth curves of patient 1 and patient 2; catch-up growth was noticed in patient 1 at 13 months and 32 months old (red circle) and in patient 2 at 10 and 14 months old (red triangle). (**f**–**i**) Sanger chromatograms of the detected variants in *NAA15*.

One intronic variant (c.1410+5G>C, Figure 1g) was predicted as a noncanonical splicing variant, so we performed the in vitro minigene splicing assay for this variant. The overlapping amplicon containing either variant or wild allele was generated by the standard PCR (see Table S1 for the primers) and cloned into the pcDNA3.1 vector. The wild-type (pcDNA3.1-wt) and mutant (pcDNA3.1-mt) vectors were transiently transfected into human breast cancer cells (MCF-7) and human embryonic kidney cells (293T) (Lipofectamine 2000 reagent, Invitrogen, Carlsbad, CA, USA) for 48 h of culture before collection. Total RNA was extracted (TRIzol, Omega, GA, USA) from transfected cells and reverse transcribed to cDNA (reverse transcription kit, SuperScript, 18064071, Thermo, Waltham, MA, USA) followed by the standard PCR. The concentration and purity of the extracted RNA were determined by UV spectrophotometry, and the PCR products were visualized on a 2% agarose gel and then purified for Sanger sequencing.

## 3. Results

### 3.1. Clinical Features and Variant Spectrum of NAA15

Four unrelated children with *NAA15* variants were detected, and all variants were novel and evaluated as likely pathogenic/pathogenic (LP/P, Table 1). Aside from *NAA15* mutation, these patients carried no other LP/P mutations.

All patients exhibited mild DD. The phenotypic findings are summarized in Table 1, and the pedigrees, physical growth curves, and Sanger traces of the detected variants are shown in Figure 1.

Patient 1 was a 13-month-old boy who presented with global development delay. He had a mild broad nasal bridge and a large forehead. At 36^+6^ weeks of gestation, he was delivered by cesarean because of intrauterine asphyxia. The Apgar scoring at birth was not available. His birth weight was 2.8 kg and birth length was 48.5 cm. He exhibited hypertonia after birth. Mild motor development delay was noted when he was 5 months old because he failed to keep the head upright. He sat independently at 8 months old, crawled at 10 months old, and murmured simple meaningless words such as “baba” and “mama” at 12 months old. His height was 75.9 cm (P25) and weight was 9.8 kg (<P50) at 13 months old. The CNBS examination revealed global developmental delay in all subscales (Table 2). We performed a follow-up study when he was 32 months old. Both height (98 cm, >P75) and weight (15.5 kg, >P75) were increased (Figure 1e), and DQ improved (Table 2). Cardiac color ultrasound and brain MRI were generally normal. One de novo missense variant (c.1321G>A, p.D441N) in *NAA15* was detected (Figure 1a,f).

Patient 2 was a 10-month-old boy. He had a mild prominent and large forehead and flat broad nose bridge. He was born at 39 weeks via normal vaginal delivery with normal weight (3.23 kg) and normal length (49 cm). His height and weight were 73.5 cm (<P50) and 8.6 kg (<P25) at 10 months of age, respectively, and increased to 80 cm (>P50) and 10 kg (>P25) at 14 months of age, respectively (Figure 1e). He came to our hospital because he failed to sit independently at 9 months of age. The ASQ evaluation at 10 months of age revealed developmental delay in both gross motor and personal–social sections (Table 2). He could crawl at the age of 12 months and stand at 14 months. He made some meaningless utterances such as “da-da” at the age of 14 months and was diagnosed with language delay. Brain MRI was normal. One de novo intron variant (c.1410+5G>C) in *NAA15* was detected, which was predicted as a noncanonical splicing variant by the dbscSNV database [16] (Figure 1b,g).

Patient 3 had been labeled as DD when she was 1 year and 5 months old. She had a mild short chin and low-set ears. She suffered from febrile convulsions twice when she was 3 years old and 5 years old. Her CNBS score before 5 years of age was not available because her medical records were in the hospital outside Beijing. The last medical interview at the age of 76 months old in our hospital showed ADHD diagnosis. Weight (18.6 kg) and height (113.5 cm) were both on P10. The CNBS revealed her general quotient was 62, and her intelligence age was 47.1 months old. One de novo nonsense variant (c.1819C>T, p.Q607X) in *NAA15* was detected (Figure 1c,h).

Patient 4 was a 2-year-old girl who was diagnosed with developmental delay and language delay. She had a mild broad nasal bridge, wide eye distance, a large mouth, and low-set ears. She was a full-term birth without suffocation; her birth weight was 3.8 kg. She could walk alone and speak a single word at the age of 19 months and speak simple words at the age of 2 years. Her DQ was 59 at the age of 20 months, and after 4 months of physical training, her DQ was 68 (Table 2). One maternal splicing variant (c.1540-1G>T) of *NAA15* was detected. No further clinical information or CNBS tests were available. The intelligence quotient and cognition status were not available for her mother (Figure 1d,i).

**Figure 2:**
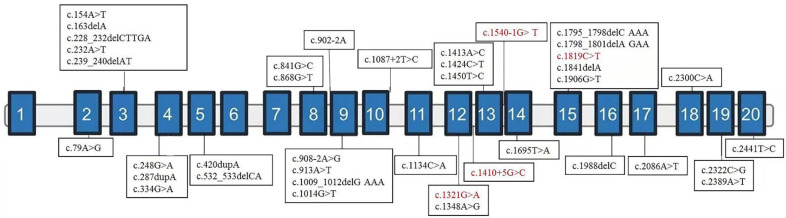
Exonic localization of *NAA15* variants. Schematic representation of the genomic structure of human *NAA15* and variants identified in this study (in red) and those previously reported elsewhere (in black). Solid rectangles indicate exons, and the horizontal bars represent introns.

After finishing the in vitro functional assay for the variant of c.1410+5G>C, these four variants were classified as likely pathogenic/pathogenic following the ACMG guidelines [15]. We retrieved another 35 variants of *NAA15* from 42 patients of previous literature together with our samples. The NDD categories of affected patients are summarized in Table 1, and the locations of variants are shown in Figure 2. *NAA15* variants are in different domains, ranging from the second to the last exon. There is no obvious correlation between exonic localization and phenotype, and 81% of affected patients carry LGD variants [11,12,17,18].

### 3.2. Minigene Splicing Assay for the Noncanonical Splice Variant (c.1410+5G>C) in NAA15

**Figure 3:**
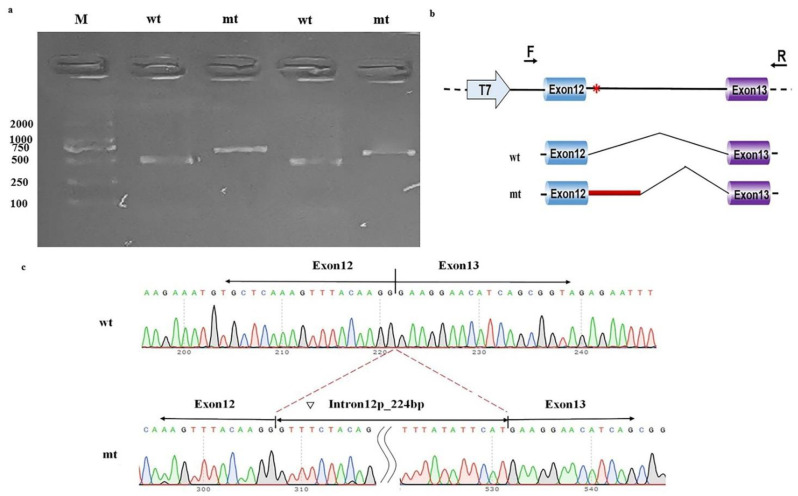
Minigene study on a noncanonical splice site variant in *NAA15*. (**a**) Reverse-transcription polymerase chain reaction (RT-PCR) products from MCF-7 (left) and HEK-293T (right) cells transfected with either wild-type (wt) or mutant (mt) pcDNA3.1 vector were separated by electrophoresis. M: DNA marker. (**b**) Structure of the splicing vector pcDNA3.1, where the symbol “*” represents the location of the variant. (**c**) Sequencing of minigene product showed normal mRNA composing exons 12 and 13 and abnormal mRNA composing 224 bps of intron 12 besides exons 12 and 13.

The in vitro functional assays of this intronic variant showed that pcDNA3.1-wt produced one unique 490 bp band in both MCF-7 cells and 293T cells, as expected. In contrast, pcDNA3.1-mt produced a larger band than pcDNA3.1-wt (Figure 3a). Sequencing and online alignment of the PCR product showed there are an additional 224 bps of intron 12 in *NAA15* (+1 to +224) at the junction of exon 12 and exon 13 (Figure 3b). These results confirmed that this intronic *NAA15* variant caused abnormal mRNA cleavage model and intron retention (Figure 3c).

## 4. Discussion

Human *NAA15* encodes a component of the NatA complex that is thought to tether the complex to the ribosome for posttranslational modification of proteins [10]. NAA15 is expressed at high levels in neonatal mouse cortex cells involving neuronal proliferation and migration [19]. In 2017, heterozygous de novo variants in *NAA15* were found to be significantly associated with NDDs from a large cohort of over 11,730 patients using a case–control design [10]. Among them, 12 LGD variants and 1 deleterious missense variant in *NAA15* were reported, producing a mutation frequency of *NAA15* of 0.11% in the NDD cohort. Previously, no Chinese patients with *NAA15* variants had been reported. In this study, three de novo *NAA15* variants and one maternal *NAA15* variant were identified from 769 Chinese NDD patients, indicating a frequency of 0.52% in a Chinese cohort (4/769).

Our four affected children presented variable neurodevelopmental features, including global DD, language and motor delay, ADHD, and seizure. More interestingly, all affected individuals herein presented mild DD based on the scores of the CNBS-R2016 or ASQ. None of them had a complaint of congenital heart disease (i.e., cyanosis, palpitations, fatigue, and recurrent respiratory infections), and normal heart development was confirmed in one patient by the cardiac color ultrasound, which is different from previous reports [11,12,17,18]. In addition, catch-up developmental trajectories were noted in three affected children based on their improved neurodevelopmental scores and physical growth curve at different ages, especially for gross motor, personal–social, and language abilities. For patient 1, the face-to-face CNBS evaluations at the ages of 13 and 37 months showed increased scores for all five subscales over 2 years after the age-matched correction (Table 2). In addition, his increased physical growth was seen in weight and height (Figure 1e). Patient 2 is not a local resident of Beijing, and the face-to-face CNBS evaluation was not available, so we compared her ASQ scores at the ages of 10 and 14 months. Patient 2 also showed increased scores on the gross motor and personal–social subscales and increased physical growth in weight and height (Table 2 and Figure 1e). Increased scores especially on the gross motor subscale were also noted in patient 4 (Table 2).

To explore the DD severity and developmental trajectory of the patients with *NAA15* pathogenic variant, we reviewed the detailed NDD phenotypes for 42 previous patients with *NAA15* variant. We found that more than half of them (26/46 = 56.5%, Table 1) had mild to moderate ID/DD (including six patients with mild ID/DD, two patients with mild/moderate ID/DD, and 14 patients with moderate ID/DD) [11,12,17,18], and possible catch-up neurodevelopment was implied in three DD patients. Cheng et al. reported a girl with a de novo variant in *NAA15* (Individual 6, Cys484Arg) who was diagnosed as “global DD at the age of 32 months” and described as “improved neurodevelopment with age grow up” [17]. Another two patients (Individual 4, Individual 10-3) in Cheng’s study showed possible catch-up developmental trajectories based on their phenotypic description: “adaptive behavior was found to be within the average range” at 9 years for Individual 4; “worked as a child-care assistant” and being a “wonderful advocate” for Individual 10-3 [12]. No relationship was found between possible catch-up development and sex, variant category (missense or LOF), or domain localization with respect to the *NAA15* variant. Although possible catch-up growth was noted in six NDD children with *NAA15* variants, due to the limitation of sample size, prospective studies with more affected children are needed to confirm the catch-up developmental trajectory of *NAA15* variants in the future.

The possible catch-up developmental trajectory has been reported in other pathogenic NDD-related variants. Platzer et al. reported the NDD phenotypes and developmental trajectories of nine children carrying de novo null variants in *CUX1*. All affected children showed developmental delay, three of whom presented normal age-matched intelligence at the ages of 4, 6, and 8 years [20]. Potential reasons explaining possible catch-up development in DD children with *NAA15* variants include the following: (a) Rehabilitation and training may improve neurodevelopmental outcome, even for the DD patients with pathogenic variants. Patients 1, 2, and 4 received discontinuous or continuous physical training after diagnosis. After physical training, patient 1 could walk at the age of 16 months, jump at the age of 29 months, and climb the stairs and express meaningful words at the age of 30 months. (b) The pathogenicity of variants with respect to NDD severity was influenced by protein expression in the brain. NAA15 is expressed at low levels during the embryonic period but is expressed at high levels after birth and at very low levels thereafter [19], suggesting that the physical role of *NAA15* in neurodevelopment decreases with the increase in age. We presumed that possible catch-up development of NDDs could occur for some certain NDD genes or some specific NDD patients, either naturally due to the diluted importance of NDD genes in brain development after birth or artificially due to the physical training in some NDD patients. With the application of clinical genomic testing, an increasing number of genetic DDs will be detected. Therefore, further follow-up studies with more NDD cases carrying different variants are needed to understand the clinical penetrance or natural developmental trajectories of different NDDs. In addition, a prospective study exploring the benefits of physical rehabilitation for genetic NDDs is needed, which is very important for precise genetic counseling and clinical management.

Haploinsufficiency of *NAA15* caused by the LOF variant has been recognized as the mechanism for this newly recognized NDD because the probability of being LOF intolerant (pLI) and the observed/expected (o/e) constraint scores for *NAA15* are 1.0 and 0.15 (0.08–0.28), respectively. One de novo deletion covering only exon 5 of *NAA15* (3.60 kb) was reported in the DECIPHER database (ID 287102) [21]. The NDD phenotypes of this single exonic deletion, including delayed speech and language development and global developmental delay, were similar to those of *NAA15* LGD variants, confirming the postulated mechanism of haploinsufficiency for *NAA15*. The pathogenicity of missense variants in *NAA15* occurring via other mechanisms cannot be excluded because the missense constraint Z-score for *NAA15* is 3.81, indicating its intolerance to missense variation [22]. Nine *NAA15* missense variants have been reported (two are inherited, six are de novo, and one is unknown) [11,17,18], and the clinical features of *NAA15* missense variants were similar to those of *NAA15* LGD variants. Unstable protein expression caused by missense variants could result in degradative protein, similar to haploinsufficiency caused by the LOF variant.

The clinical pathology interpretation of noncanonical splicing variants is a major challenge for diagnostic laboratories because most of these variants are outside essential splice sites, offering low-precision prediction value in the context of bioinformatic predictions. The guidelines from the ACMG state that the functional evidence from RNA analysis can be used for variant interpretation [15]. One study also pointed out that blood RNA analysis can increase pathogenic evidence and resolve pathogenic reclassification for 33% of splicing variants [23]. However, successful RNA analysis depends on the quality of blood and alternative expression of target genes in blood. In this study, we detected a noncanonical splicing variant of *NAA15* (c.1410+5G>C). No databases showed direct evidence of high *NAA15* expression in blood. Therefore, we performed an in vitro splicing assay and illuminated that this variant created one cryptic donor splice site and resulted in intron retention. Jenny Lord et al. estimated that 27% of pathogenic splicing variants within the Deciphering Developmental Disorders cohort are in noncanonical positions [24]. Another study based on 689,321 clinical genomic tests showed that splicing variants account for 13% of clinically reportable variants, and 5.4% of them were labeled as variants of uncertain significance (VUS). With the help of RNA analysis, 1.7% of tested individuals were reclassified, improving the overall diagnosis for 0.1% of tested individuals [25]. Our study proved that functional experiment platforms of clinical diagnostic laboratories will help to interpret the noncanonical splicing variants.

## 5. Conclusions

In conclusion, our study expanded the variant spectrum of *NAA15* using clinical and genetic information from Chinese NDD patients. The affected patients presented mild DD and possible catch-up developmental trajectories, especially in gross motor, personal–social, and language abilities. Prospective studies are needed to understand the clinical presentations and prognosis of patients with these kinds of disorders.

## Figures and Tables

**Table 1:** Clinical and genetic information of patients with *NAA15* variants.

	Patient 1	Patient 2	Patient 3	Patient 4	Literature	Total	Percent
Age (months)	13	10	17	24			
Nucleotide change *	c.1321G>A	c.1410+5G>C	c.1819C>T	c.1540-1G>T			
Genomic location	Chr4:140280960	Chr4:140281054	Chr4:140291430	Chr4:140282877			
Amino acid change	p. D441N	−	p. Q607X	−			
Inheritance	de novo	de novo	de novo	maternal			
ACMG classification	LP	LP	P	LP			
Developmental delay (DD)	+	+	+	+	10/40	13/43	23.3
Gross motor delay	+	+	+	+			
Fine motor delay	+	−	+	−			
Language delay	+	+/−	+	+			
Personal–social behavior delay	+	+	+	+			
Adaptive behavior delay	+	−	+	+			
Mild/moderate DD	+	+	+	+	22/42	26/46	56.5
ADHD or behavioral issues	−	−	+	+	35/41	37/45	82.2
Seizures	−	−	+	−	10/33	11/36	30.6
Abnormal brain MRI	−	−	−	−	3/14	3/17	17.6
Muscle tone issue	−	+	−	−	9/24	10/27	37.0

* NM_057175.4 (on GRCH37/hg19 assembly). +: positive/present; −: negative/absent; N/A: not available. ADHD: attention deficit hyperactivity disorder; P: pathogenic; LP: likely pathogenic; OFC: occipitofrontal circumference.

**Table 2:** Neurodevelopmental scores of patients with *NAA15* variants.

	Age	Assessment Tool	Score				
			Gross Motor	Fine Motor	Language	Personal–Social	Adaptive Behavior
Patient 1	13 month	CNBS	64	67	60	60	67
	37 month	CNBS	104	72	85	77	77
Patient 2	10 month	ASQ	5	55	30	25	50
	14 month	ASQ	35	45	30	45	50
Patient 3	76 month	CNBS	59	63	63	65	59
Patient 4	20 month	CNBS	62	57	58	56	63
	24 month	CNBS	80	62	65	62	69

## Data Availability

All data generated or analyzed during this study are included in this published article and its supplementary information files.

## Supplementary Materials

The following supporting information can be downloaded at: https://www.mdpi.com/article/10.3390/genes13030536/s1, Table S1: The primers used in the functional study of c.1410+5G>C in *NAA15*.
