# Supplementary material for: Possible Catch-Up Developmental Trajectories for Children with Mild Developmental Delay Caused by NAA15 Pathogenic Variants
Source: Genes (Basel). 2022 Mar 18;13(3):536. doi: 10.3390/genes13030536 (PMC8954815; doi:10.3390/genes13030536)
Supplement: Supplementary file 1 [file genes-13-00536-s001.zip › genes-1633905-supplementary.pdf]

Supplementary Table S1 The primers used in the functional study of c.1410+5G>C in *NAA15*

primers	primer sequence（5’- 3’）
56687-NAA15-F	cagattgtaggcatgagccc
56995-NAA15-F	cgtacagatttggctatgcc
59174-NAA15-R	ggtcctaggagtactttaat
59470-NAA15-R	agtaagtcaagcaatcagga
pcDNA3.1-NAA15-KpnI-F	GCTTGGTACCATGCATGCTGGAAATATTAAAGA
pcDNA3.1-NAA15-BamHI-R	TAGTGGATCCTCTCTCAATCTCATGACATT
